# Sentinel lymph node biopsy before and after neoadjuvant chemotherapy in cN0 breast cancer patients: impact on axillary morbidity and survival—a propensity score cohort study

**DOI:** 10.1007/s10549-024-07274-1

**Published:** 2024-04-18

**Authors:** Sergi Fernandez-Gonzalez, Catalina Falo, Maria J. Pla, Miriam Campos, Carlos Ortega-Exposito, Raul Ortega, Maria Vicente, Ana Petit, Jan Bosch-Schips, Maria Teresa Bajen, Gabriel Reyes, Evelyn Martínez, Javier González-Viguera, Judith Peñafiel, Agostina Stradella, Sonia Pernas, Jordi Ponce, Amparo Garcia-Tejedor

**Affiliations:** 1 Department of Gynecology, Multidisciplinary Breast Cancer Unit, Hospital Universitari Bellvitge, Idibell, c/ Feixa Llarga s/n. Hospitalet de Llobregat, CP: 08907 Barcelona, Spain; 2 Facultat de Medicina i Ciències de la Salut, Universitat de Barcelona (UB), Feixa Llarga, s/n, 08907 l’Hospitalet de Llobregat, Spain; 3 Department of Medical Oncology, Multidisciplinary Breast Cancer Unit, Institut Català d’Oncologia, Idibell, Barcelona, Spain; 4 Department of Radiology, Multidisciplinary, Breast Cancer Unit. Hospital Universitari Bellvitge, Idibell, Barcelona, Spain; 5 Department of Pathology, Multidisciplinary Breast Cancer Unit, Hospital Universitari Bellvitge, Idibell, Barcelona, Spain; 6 Department of Nuclear Medicine, Multidisciplinary Breast Cancer Unit, Hospital Universitari Bellvitge, Idibell, Barcelona, Spain; 7 Department of Radiation Oncology, Multidisciplinary Breast Cancer Unit, Institut Català d’Oncologia, Barcelona, Spain; 8 Biostatistics Unit, Institut d’Investigació Biomèdica de Bellvitge, Hospitalet de Llobregat, Spain

**Keywords:** Breast cancer, Neoadjuvant chemotherapy, Sentinel lymph node biopsy, Axillary lymph node dissection

## Abstract

**Purpose:**

In patients with clinically lymph node-negative (cN0) breast cancer, performing sentinel lymph node biopsy (SLNB) after neoadjuvant chemotherapy (NACT) has been preferentially embraced in comparison to before NACT. However, survival outcomes associated with both strategies remain understudied. We aimed to compare the axillary lymphadenectomy (ALND) rate, disease-free survival (DFS), and overall survival (OS), between two strategies.

**Methods:**

We included 310 patients in a retrospective observational study. SNLB was performed before NACT from December 2006 to April 2014 (107 cases) and after NACT from May 2014 to May 2020 (203 patients). An inverse probability of treatment weighting (IPTW) method was applied to homogenize both groups. Hazard ratios (HR) and odd ratios (OR) are reported with 95% confidence intervals (95%CI).

**Results:**

The lymphadenectomy rate was 29.9% before NACT and 7.4% after NACT (*p* < 0.001), with an OR of 5.35 95%CI (2.7–10.4); *p* = .002. After 4 years of follow-up, SLNB after NACT was associated with lower risk for DFS, HR 0.42 95%CI (0.17–1.06); *p* = 0.066 and better OS, HR 0.21 CI 95% (0.07–0.67); *p* = 0.009 than SLNB before NACT. After multivariate analysis, independent adverse prognostic factors for OS included SLNB before NACT, HR 3.095 95%CI (2.323–4.123), clinical nonresponse to NACT, HR 1.702 95% CI (1.012–2.861), and small tumors (cT1) with high proliferation index, HR 1.889 95% (1.195–2.985).

**Conclusion:**

Performing SLNB before NACT results in more ALND and has no benefit for patient survival. These findings support discontinuing the practice of SLNB before NACT in patients with cN0 breast cancer.

**Supplementary Information:**

The online version contains supplementary material available at 10.1007/s10549-024-07274-1.

## Introduction

Sentinel lymph node biopsy (SLNB) has been proven equally safe and accurate compared to axillary lymph node dissection (ALND) [1, 2] when evaluating axillary status in cN0 patients, while also benefiting from less morbidity and better quality of life outcomes for patients [3]. Until 2015, performing SLNB before NACT was recommended as it enables the basal assessment of nodal status [4]. However, SLNB before NACT has been associated with higher rates of positive SLNB and consequently, higher rates of ALND [5]. In addition, with evidence that 22% to 35% of patients will have no residual disease in the axilla after NACT [6, 7], this raises the question of whether ALND could have been avoided.

Multiples studies in the last decade have reported similar identification rates and false-negative rates (FNRs) when performing SLNB before and after NACT [8, 9]. Regarding the latter, a recent meta-analysis found an identification rate of 94% (95%CI, 92–96) and an FNR of 7% (95%CI, 5–9) [10]. Additional advantages, such as the assessment of “in vivo” tumoral response, prompt initiation of NACT, and the simultaneous performance of both SNLB and breast surgery, are associated with SLNB after NACT. Consequently, this timing has been preferentially embraced in comparison to before NACT but the latter still remains optional in accordance with the latest guidelines [11–13].

Although the advantages and disadvantages of performing SLNB before or after NACT are well known in patients with cN0 disease, comparison of the long-term oncological survival outcomes remains understudied to date. To provide results in survival outcomes following SLNB before and after NACT, it may help the scientific community to homogenize clinical practice [14, 15]. This study aimed to compare ALND rates when SLNB is performed before and after NACT. The impact of these strategies on disease-free survival (DFS) and overall survival (OS) are then assessed in a series of breast cancer patients’ cN0 disease treated by NACT after 4 years of follow-up.

## Methods

### Study design and participants

Consecutive patients with breast cancer and negative axillary lymph nodes (cN0) from our Breast Cancer Unit (Hospital Universitari Bellvitge & Institut Català d’Oncologia) who were candidates for NACT were enrolled for retrospective analysis from a prospectively maintained database. Patients were divided into two cohorts. Cohort A included patients who underwent SLNB before NACT between December 2006 and April 2014, whereas Cohort B included patients who underwent SLNB after NACT between May 2014 and May 2020. Patients were required to meet all the inclusion criteria: age 18–80 years old; negative axillary lymph nodes after ultrasound assessment, core biopsy showing an infiltrating breast carcinoma; stage > cT1b; and candidates for chemotherapy before surgery (according to our multidisciplinary committee). Exclusion criteria were age > 80 years, pathologically confirmed clinically positive axillary lymph node and a personal history of ipsilateral breast cancer. The ethics committee of our hospital approved the study (247/06). All patients gave written informed consent for NACT and surgical procedures. No large language models were used in the preparation of this manuscript.

### Treatment protocol

Between 18 and 24 h before surgery, 3 mCi 99mTc-albumin nanocolloids were injected in breast for lymphatic mapping. Intraoperative gamma probe (Europrobe; Britec; Sheffield; UK) was used for SLN identification. The SLN before NACT performed between 2007 and 2011 and all SLN after NACT were processed histologically. They were cut into 1–2-mm-thick slices and were formalin fixed and paraffin embedded. From each slice, 6 consecutive series of microtome sections with gaps of 150 µm between them were prepared. They were stained alternatively by Hematoxilin-eosin and AE1–AE3 cytokeratin (DAKO IR053/IS053) immunohistochemistry. However, the SLN before NACT between 2011 and April 2014 was studied by one-step nuclei acid amplification (OSNA) [16]. Isolated tumor cells (ITCs) were defined as small clusters of cells not greater than 0.2 mm or single tumor cells, or a cluster of fewer than 200 cells in a single histologic cross section by HE/IHC or less than 160 copies by OSNA. Micrometastasis was defined as a small tumor cluster of cells measuring 0.2–2 mm by HE/IHC or 251–5000 copies by OSNA. Macrometastasis was defined for values greater than either 2 mm by HE/IHC or more than 5000 copies by OSNA.

NACT included anthracyclines followed by taxanes for 6 months, and adding trastuzumab in HER2-positive tumors. Since October 2016, pertuzumab was added to trastuzumab and chemotherapy in patients with human epidermal growth factor receptor (HER2)-positive tumors. Breast conservative or radical surgery was decided considering response to NACT, tumor size, predictable esthetic results, and initial tumor characteristics. Complete ALND was performed only when macrometastasis was identified in the SLNB. Patients with negative SLNB, isolated tumoral cells, or micrometastasis did not undergo further axillary treatment. During the time period covered by this study, the indication for axillary lymphadenectomy has remained consistent in both time periods that is for macrometastasis in the sentinel node. Adjuvant radiotherapy was indicated whether breast-conserving surgery, tumoral margins affected or ink margins, T3–T4 tumors, and/or positive SLNBs. Endocrine therapy was indicated in receptor hormonal-positive tumors. Trastuzumab was completed up to one year in those HER2-positive ones according to international guidelines. Since 2017, in patients with TN and residual invasive disease following tumors NACT, 6–8 cycles of Capecitabine were added [17].

Pathological complete response (pCR) was defined as the absence of invasive carcinoma in both the tumor bed and the axillary nodes with a 100% of fibrosis replacing the invasive carcinoma (ypT0 or ypTis). Pathological partial response was characterized by the pathologist observing more than 30% fibrosis replacing the infiltrating carcinoma, while no response was indicated when fibrosis was less than 30%.

### Data collection

Clinical and pathologic variables of interest included age, tumor size before and after NACT, histological type, Nottingham histological grade, estrogen and progesterone receptors (ER, PR), immunohistochemical expression, HER2 status, Ki67 proliferative index, and the sentinel lymph node identification rate and involvement. The clinical imaging-based tumor response was classified according to the WHO criteria [18]. Cases were classified according to immunohistochemical determination of ER, PR, and HER2 status into four subtypes [19, 20]: ER + /HER2(−), ER + /HER2 + , ER(−)/HER2 + , and Triple Negative (ER(−)/HER2(−)). Disease-free survival (DFS) was defined as the time from starting NACT to disease recurrence or death due to any cause, and overall survival (OS) was defined as the time from starting NACT to death due to any cause.

### Statistical analyses

A descriptive analysis was performed to characterize clinical profile of subjects included in this study. Mean and standard deviation were used for continuous variables, and median and interquartile range for those variables with a non-normal distribution. The number of cases and percentages were presented for categorical variables.

To assess heterogeneity in baseline characteristics between the timing of sentinel node removal (SLN pre-NACT and post-NACT), we used absolute standardized differences, defined as the absolute standardized mean difference for continuous variables and the absolute raw difference for categorical variables. A covariate was considered heterogeneous between groups if the absolute standardized differences were > 0.1. The inverse probability of treatment weights (IPTW) approach was used to create a pseudo-population in which the groups were balanced on baseline characteristics (IPTW dataset). Stabilized weights were calculated using propensity scores obtained from logistic regression adjusted for age, T stage at diagnosis, breast cancer subtype, menopausal status, and clinical imaging response. To assess heterogeneity in baseline characteristics between groups in the IPTW dataset, absolute standardized differences were also calculated (Fig. 2). After IPTW balancing, all covariates were homogeneous (< 0.1).

The Kaplan–Meier method was used to estimate probability of survival, while log-rank test was applied to compare groups. The relative risk and 95% confidence intervals (CI) were estimated to compare DFS and OS between groups. Cox-proportional hazards models were used to calculate hazard ratios (HR) and the 95% CI of each prognostic factor by univariate and multivariate analyses. All these analyses were performed in the original dataset and including propensity score calculated with IPTW method.

All of the analyses were performed with the statistical software package R version 4.1.0 for Windows (http://www.R-project.org, The R Foundation).

## RESULTS

**Fig. 1:**
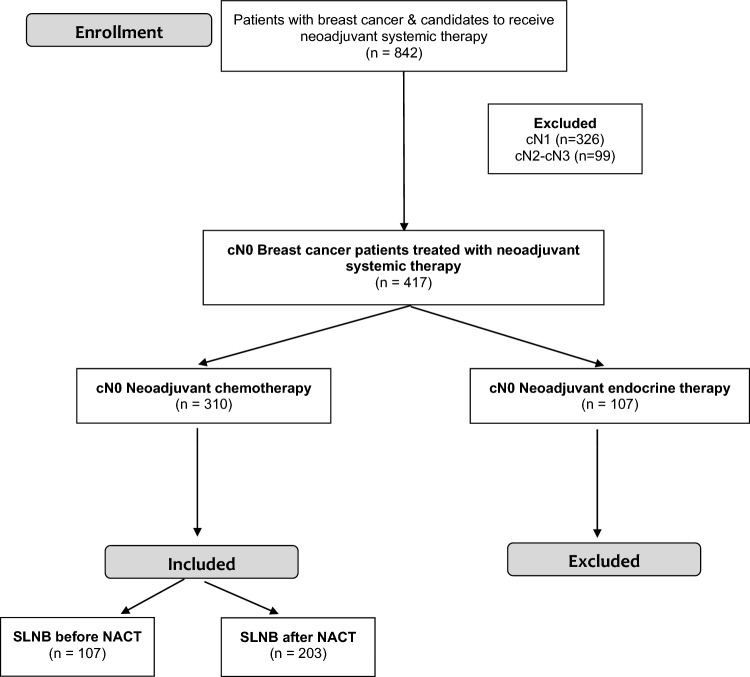
Flow chart

**Fig. 2:**
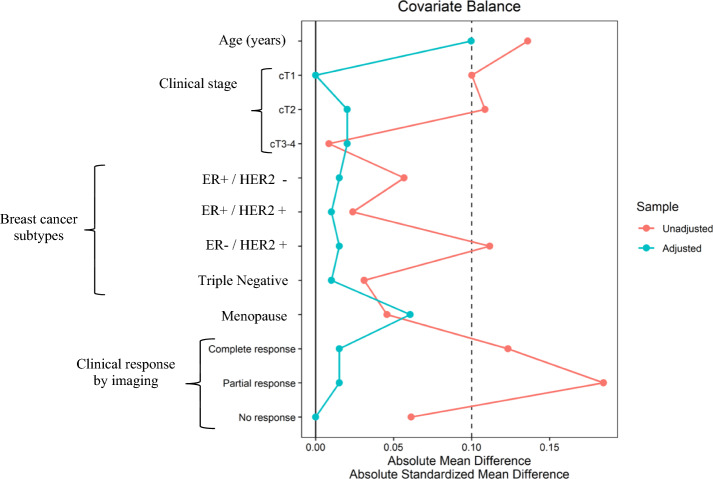
Baseline characteristics before and after balancing by IPTW

In total, data were collected for 310 patients (Fig. 1) and divided into Cohort A (SLNB before NACT) and Cohort B (SLNB after NACT) with 107 and 203 patients, respectively. The clinical characteristics of patients with an absolute mean or standardize difference > 0.1 between groups were considered covariates unwell balanced. After applying IPTW balancing, all covariates show < 0.1 absolute mean or standardize difference, and they were considered well-balanced covariates (Fig. 2). Results are shown in Table 1 before and after the IPTW analysis.

**Table 1:** Results of clinical characteristics and distribution of breast cancer subtypes

	Original sample		IPTW data	
	pre-NACT SLN (*n* = 107)	post-NACT SLN (*n* = 203)	pre-NACT SLN (*n* = 103.82)	post-NACT SLN (*n* = 202.94)
Age (mean, years, SD)	49.9 ± 12.7	51.6 ± 12.6	50.61 ± 13.02	50.94 ± 12.46
Menopausal status (%)	51 (47.7)	106 (52.2)	52.3 (50.4)	103.1 (50.8)
T stage at diagnosis (%)				
cT1	1 (0.09)	23 (11.3)	4.8 (4.7)	15.7 (7.7)
cT2	86 (92)	152 (74.9)	83.7 (80.6)	158.9 (78.3)
cT3	14 (13.1)	24 (11.8)	15.3 (14.7)	28.4 (14)
cT4	0	4 (2)	1.6 (1.5)	3.3 (1.6)
Histological subtype (%)				
Invasive ductal carcinoma	103 (96.3)	185 (91.1)	- -	
Invasive lobular carcinoma	4 (3.7)	12 (5.9)	–	–
Nottingham grade (%)				
G1	3 (2.8)	6 (3)		–
G2	38 (35.5)	65 (32)		–
G3	66 (61.7)	132 (65)		–
Breast cancer subtype ** (%)				
Hormone receptor + Her2−	28 (26.2)	64 (31.5)	30 (28.9)	60 (29.6)
Hormone receptor + Her2 +	23 (21.5)	49 (24.1)	21.4 (20.5)	45.6 (22.5)
HER2 pure	21 (19.6)	17 (8.4)	16.1 (15.5)	26.8 (13.2)
Triple negative	35 (32.7)	73 (36)	36.4 (35)	70.6 (34.8)
Clinical response by imaging				
Complete response	51 (47.7)	73 (36)	37.7 (36.3)	80.3 (39.6)
Partial response	42 (39.3)	116 (57.1)	56.2 (54.2)	103. (50.9)
No response	14 (13.1)	14 (6.9)	9.9 (9.5)	19.4 (9.5)

*IPW* Inverse probability weighting, *pre-NACT SLN* Sentinel Lymph Node performed before chemotherapy treatment, *post-NACT SLN* Sentinel Lymph Node performed after chemotherapy treatment. **Hormone receptor + HER2 -: ER + and Ki67 < 20% and HER2 -. Hormone receptor + HER2 + : ER + and Ki67 ≥ 20% and HER2 + . HER2 + : ER—and PR—and HER2 + . Triple Negative: ER—and PR—and HER2 -

Figure 2 A covariate was considered heterogeneous between groups if the absolute standardized differences were > 0.1. The inverse probability of treatment weights (IPTW) approach was used to create a pseudo-population in which the groups were balanced on baseline characteristics (IPTW dataset). After IPTW balancing, all covariates were homogeneous (< 0.1)

The SLN identification rate was 100% (107 cases) in the pre-NACT group and 97.5% (198 out of 203 cases) in the post-NACT group, without statistical significance difference. However, sentinel lymph node positivity rates differed significantly at 42.1% for SLNB before NACT and 12.5% for SLNB after NACT (*p* = 0.0001; Table 2). The lymphadenectomy rate for SLNB before NACT was 29.9% (32 patients) compared with 7.4% (15 patients) for SLNB after NACT (*p* < 0.001; Table 2). The odds ratio (OR) for performing lymphadenectomy before NACT was 5.35 (95%CI, 2.7–10.4; *p* = 0.002). Furthermore, the rates of achieving complete response in both the breast and axilla (ypT0–N0) were 24.3% (26 patients) when SLNB was before NACT and 33.5% (68 patients) when it was after NACT (*p* = 0.094). A detailed analysis of all pathological responses is shown in the Supplementary Information (Figure SI-1) and rates of positive SLN according breast cancer subtype are also shown in Supplementary Information (Table SI-1).

**Table 2:** Surgical and adjuvant treatment

	pre-NACT SLN (*n* = 107)	post-NACT SLN (*n* = 203)	*p* value
Dx-NAT time* (median, range), days	25 (13–92)	24 (5–64)	0.456
Breast conservative surgery (%)	81 (75.7)	141 (69.5)	0.436
SLN identification rate (%)	107 (100)	198 (97.5)	0.675
Patients with SLN positive (%)	45 (42.1)	25 (12.5)	**0.001**
Macrometastases (%)	31 (68.9)	11 (44)	
Micrometastases (%)	14 (32.1)	14(46)	
SLN removed (median, range)	2 (1–6)	2 (0–6)	0.158
Lymphadenectomy (%)	32 (29.9)	15 (7.4)	** < 0.001**
LN removed (median, range)	15 (6–27)	15 (10–31)	0.334
No residual disease (%)	21 (65.6)	8 (53.3)	0.419
Radiotherapy (%)			0.352
Breast RT	81 (75.7)	143 (70.4)	
Nodal RT	24 (22.7)	27 (13.3)	
Adjuvant treatment (%)			
Chemotherapy	8 (7.5)	33 (16.3)	0.03
Endocrine	54 (50.5)	117 (57.6)	0.228

* Time between diagnosis and systemic therapy. *SLN* Sentinel Lymph Node

**Fig. 3:**
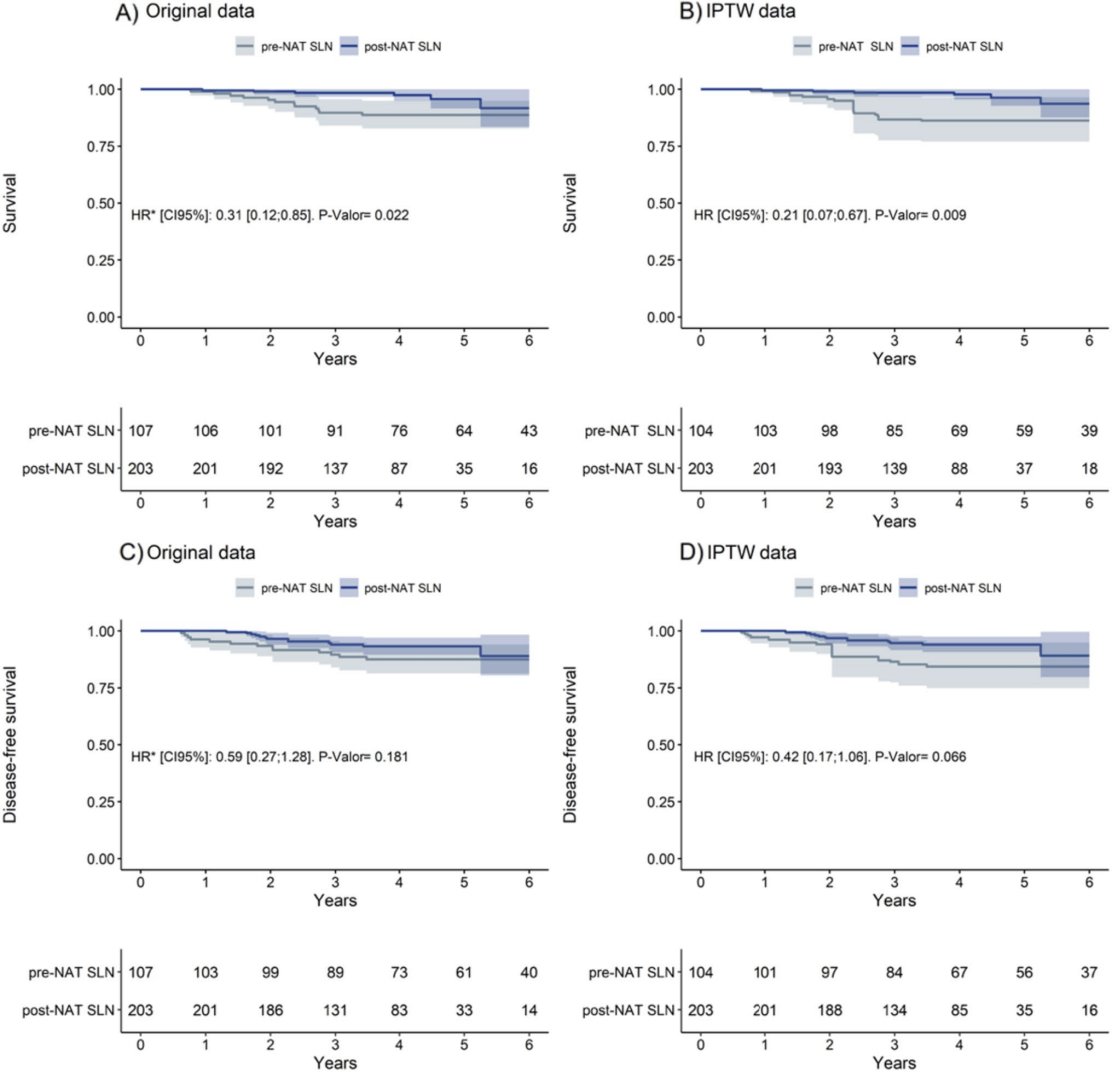
Overall Survival (Survival) and Disease-free survival in original data and after IPTW

Median follow-up periods were 64 months (range, 12–164 months) for SLNB before NACT and 44 months (range, 7–97 months) for SLNB after NACT. The 4-year OS rates were as follows: 88.4% (95%CI, 82.3–94.6; 12 deaths) for SLNB before NACT and 95.7% (95%CI, 91.5–99.9; 5 deaths) for SLNB after NACT. After applying IPTW to balance the groups, SLNB after NACT was associated with a significantly better OS for any cause of death (HR, 0.21; 95%CI, 0.07–0.67; *p* = 0.009), as shown in Fig. 3B.

The 4-year DFS rates were 87.5% (95%CI, 81.2–93.9; 13 events) for SLNB before NACT and 93.1% (95%CI, 89.3–97; 12 events) for SLNB after NACT. Following IPW balancing, SLNB after NACT was associated with a reduced risk of disease recurrence or death from any cause compared to SLNB before NACT (HR, 0.42; 95%CI, 0.17–1.06; *p* = 0.066), as shown in Fig. 3D. Local or systemic recurrence occurred in 12.1% for SLNB before NACT and 6.5% for SLNB after NACT (*p* = 0.083). Table 3 details the patterns of recurrence and cause of death.

**Table 3:** Pattern of recurrences

	pre-NACT SLN (*n* = 107)	post-NACT SLN (*n* = 203)	*p* value
No recurrence (%)	94 (87.9)	190 (93.5)	0.083
Local recurrences (%)	1 (0.9%)	4 (2)	
Axillary (%)	0	0	
Systemic recurrence (%)	12 (11.2)	9 (4.4)	
Deaths from breast cancer (%)	13 (12.1)	4 (2)	0.001
Deaths from any cause (%)	16 (14.9)	6 (2.9)	0.001

In pre-NACT 2/12 patients experienced local + distant recurrence

In post-NACT: 5/ 9 patients experienced local + distant recurrence

Figure 3 Kaplan–Meier curves for overall survival (Survival) and disease-free survival in original and IPW datasets are presented. Post-NACT group was associated with better OS for any cause of death (HR, 0.21 95%CI, 0.07–0.67; *p* = 0.009) and better DFS, HR 0.42 CI (0.17–1.06) (*p* value 0.066) (Fig. 3D).

Multivariate analysis for prognostic factors of OS revealed that T1 stage, no response to NACT, and SLNB before NACT significantly and negatively affected OS (Table 4). T1 stage and SLNB before NACT remained independent factors contributing to a worse DFS. In the global cohort, there were no significant differences in DFS by SLNB involvement (Figure SI-2). Sensitivity statistical analysis is shown in figure SI-3 after the exclusion of 44 patients in post-NACT group, 9 for receiving Capecitabine and 35 for receiving trastuzumab + Pertuzumab. Post-NACT group was associated with better OS for any cause of death (HR, 0.19 95%CI, 0.06–0.65; *p* = 0.008) and better DFS, HR 0.45 CI (0.18–1.13) (*p* value 0.087).

**Table 4:** Multivariate analysis of Prognostic Factors for Overall Survival (OS) and Disease-Free Survival (DFS)

Characteristic	OS and HR (95%CI)	P value		DFS and HR (95%CI)		P value
Menopausal status						
Pre-menopause	Ref			Ref		
Menopause	1.064 (0.82–1.381)	0.641		1.028 (0.788–1.341)		0.837
T stage at diagnosis (%)						
cT1	1.889 (1.195–2.985)	0.006		2.037 (1.287–3.223)		0.002
cT2	.Ref			Ref		
cT3	1.373 (0.946–1.993)	0.095		1.232 (0.886–1.702)		0.218
cT4	2.56 (0.802–8.173)	0.112		2.94 (0.916–9.432)		0.07
Breast cancer subtype ** (%)						
Hormone receptor + Her2−	Ref			Ref		
Hormone receptor + Her2 +	1.07 (0.77–1.487)	0.688		1.228 (0.886–1.702)		0.218
HER2 pure	0.66 (0.433–1-008)	0.055		0.613 (0.399–0.943)		0.026
Triple negative	0.813 (0.602–1.097)	0.176		0.776 (0.571–1.056)		0.107
Clinical response by imaging						
Complete response	Ref			Ref		
Partial response	1.58 (0.961–2.598)	0.071		1.456 (0.897–2.364)		0.128
No response	1.702 (1.012–2.861)	0.045		1.442 (0.866–2.401)		0.160
SLNB timing						
pre-NACT SLN	3.095 (2.323–4.123)	< 0.001		2.788 (2.095–3.709)		< 0.001
post-NACT SLN	Ref			Ref		
SLNB results						
Negative	Ref			Ref		
Macrometastasis	0.506 (0.14–1.829)	0.299		0.699 (0.184–2.651)		0.598
Micrometastasis	1.172 (0.722–1.9)	0.521		1.06 (0.653–1.721)		0.813
Isolated tumor cells	0.760 (0.247–2.392)	0.65		0.969 (0.311–3.018)		0.957
Breast pathological response						
Complete	Ref					
Partial	0.896 (0.644–1.245)	0.511		0.971 (0.695–1.356)		0.863
No response	0.952 (0.636–1.424)	0.810		1.03 (0.684–1.551)		0.887
Breast radiotherapy						
No	Ref			Ref		
Breast radiotherapy	0.841 (0.601–1.178)	0.314		0.787 (0.57–1.087)		0.146
Breast + chest wall	1.466 (0.823–2.611)	0.194		1.326 (0.795–2.212)		0.279
Axillary radiotherapy						
No	Ref			Ref		
Supraclavicular and level III	1.098 (0.58–2.078)	0.774		1.09 (0.569–2.088)		0.795
Axilla + supraclavicular + level III	1–224 (0.67–2.234)	0.511		1.335 (0.733–2.431)		0.345

*OS* Overall Survival, *DFS* Disease-free Survival, *Pre-NACT SLN* Sentinel Lymph Node performed before chemotherapy treatment**,** *post-NACT SLN*: Sentinel Lymph Node performed after chemotherapy treatment. **Hormone receptor + HER2 -: ER + and Ki67 < 20% and HER2 -. Hormone receptor + HER2 + : ER + and Ki67 ≥ 20% and HER2 + . HER2 + : ER—and PR—and HER2 + . Triple Negative: ER—and PR—and HER2 -

## Discussion

In this study, we observed that patients with cN0 breast cancer who underwent SLNB after NACT had lower rates of ALND and improved DFS and OS rates compared with patients who underwent SLNB before NACT. According to our knowledge, this first IPTW study design to evaluate oncological outcomes before and after NACT found no clinical benefit when performing SLNB before NACT based not only on the similar recurrence and mortality rates but also on the higher rates of axillary lymph node dissection compared to performing SLNB after NACT. Greater ALND-associated morbidity has already been established in previous studies by our group [21]. This is further supported by the results of a meta-analysis published by Bromham N et al. [22], who demonstrated that compared to ALND, patients undergoing SLNB had a lower risk of lymphedema after < 12 months (OR, 0.33; 95%CI, 0.23–0.47; *p* < 0.0001), a lower risk of paresthesia (OR, 0.15; 95%CI, 0.09–0.23; *p* < 0.0001), and a lower risk of arm numbness (OR, 0.43; 95%CI, 0.34–0.54; *p* < 0.0001).

For almost a decade, the detection rate and FNR were major concerns when assessing SLNB after NACT. In 2022, three meta-analyses were published that offered more evidence on these issues for patients with cN0 and cN + breast cancer when comparing single and dual tracers [23–25]. In cN0 disease, the sentinel node detection rate exceeded 95% with no significant differences between single- and dual-tracer mapping: blue dye only, 96% (95%CI, 91–100); radiocolloid only, 96% (95%CI, 94–99); and both blue dye and radiocolloid, 97% (95%CI, 96–98) [26]. Those results are consistent with our data showing a detection rate of 97.5% for SLNB after NACT and 100% for SLNB before NACT (not significant) with radiocolloid. By contrast, a recent meta-analysis reported that the sentinel node FNR was 7%–12% after NACT [23–25] compared to 10%–13% before NACT [26, 27]; consequently SLNB is an accurate method for assessing axillary status after NACT. SLNB is, therefore, an accurate method for assessing axillary status after NACT. Although we could not calculate the FNR, our sentinel node-positive rates among patients after NACT were 12.5% (25/203) compared with 42.1% (45/107) before NACT, which agrees with existing data [28, 29].

Axillary recurrences were not observed with SLNB performed either before or after NACT in this study. Similarly, after 36 months of median follow-up, the GANEA2 study only reported one case of axillary recurrence among 419 patients with cN0 disease [30]. Interestingly, our multivariate analysis also showed that findings of macrometastasis or micrometastasis results on SLNB were not independent risk factors for a worse prognosis in our select sample of patients with cN0 breast cancer receiving NACT. However, patients who underwent SLNB before NACT had worse OS (HR, 3.098; 95%CI, 2.323–4.123; *p* < 0.001) and worse DFS (HR, 2.788; 2.095–3.790; *p* < 0.001) compared with those who underwent SLNB after NACT. The risk of macrometastasis at SLNB in patients with cN0 breast cancer decreased markedly after NACT in our series. We observed macrometastasis in 28% and micrometastasis in 13.1% when performing SLNB before NACT, versus macrometastasis in 5.5%, micrometastasis in 14%, and isolated tumor cells in 2.5% when performing SLNB after NACT. Kaplan–Meier analysis identified no significant differences between negative SLNB, macrometastasis, or micrometastasis in either study cohort. This can be explained by the small sample size (few micrometastases), lack of follow-up, or higher impact of molecular subtype on prognosis (rather than SLNB results) in cN0 breast cancer. In contrast, Wong et al. [31] from Dana-Farber Cancer Institute in boston (MA) analyzed 967 patients retrospectively and observed that patients with SLNypN1mi/i( +) have a worse prognosis compared to those with SLNypN0 over 5 years of median follow-up. They found a proportional decrease in survival related to the amount of residual disease in the lymph nodes after NACT, indicating 5-year OS of 88.8%, 82.8%, 79.5%, and 77.6%, for ypN0, ypNo(i +), ypN1mic, and ypN1, respectively.

The role of ALND in the presence of a macrometastasis at SLNB after NACT appears to be well defined, but uncertainties persist regarding the potential benefits of performing axillary lymphadenectomy for SLNypN1mi/i( +). This question is expected to be addressed by Tinterri et al. in the ongoing NEONOD 2 [32]. In this regard, the IBCSG 23–01 trial [33, 34] has already demonstrated no clinical benefit in terms of OS or DFS when performing ALND in patients with early-stage breast cancer with ≥ 1 micrometastasic sentinel node. This rationale was adopted in our institution, and it was applied in patients with cN0 disease who received NACT. Note that, only 39% of the panelists in the last St. Gallen consensus [12] voted in favor of performing ALND for micrometastasis and instead suggested axillary radiotherapy as valid alternative for these patients. At the present, the standard of care entails conducting ALND upon encountering macrometastasis in the post-neoadjuvant SLNB. However, there are two ongoing studies where patients who have SLNB positive after NACT are being randomized to receive either ALND or axillary radiotherapy. The results of the Alliance A011202 and the ADARNAT trial NCT04889924 [35] will be crucial to determine whether radiotherapy is non-inferior to ALND in terms of DFS and OS in patients with a positive SLNB after NACT. Research is ongoing in two trials: the Alliance A011202 and the ADARNAT trial NCT04889924.

Despite being limited to a retrospective analysis, we used clear objectives and a good quality of the study design may overcome the retrospective nature of the present study. By balancing the two study cohorts using IPTW, we could reduce the bias introduced by confounders and to evaluate whether SLNB performed before or after NACT-affected survival. On the other hand, the improvement over time in the standard of care may positively influence better outcomes in the earlier cohort. However, this fact does not impact the reduction of axillary lymphadenectomies occurring in this group. After the exclusion of those patients who received latest systemic therapies such as Capecitabine and Trastuzumab, we still observe better oncological outcomes after IPTW sensitivity analysis. Consistent with our results, performing the SLNB technique after NACT carries a reduced risk of ALND, and at the very least, provides equivalent OS and DFS.

## Conclusion

In conclusion, performing SLNB after NACT is associated with less axillary lymphadenectomy, lower recurrences rate, and no detrimental effect on survival. Therefore, in the absence of no clinical benefit, our results indicate that SLBV should no longer be recommended before NACT in patients with clinically node-negative breast cancer.

### Supplementary Information

Below is the link to the electronic supplementary material.

Supplementary file1 (DOCX 309 kb)

## Data Availability

The datasets generated during the current study are no publicly available due to protect study participant privacy but they are available from the corresponding author on reasonable request.

## Abbreviations

cN0: Clinically lymph node negative
SLNB: Sentinel lymph node biopsy
NACT: Neoadjuvant chemotherapy
ALND: Axillary lymphadenectomy
DFS: Disease-free survival
OS: Overall survival
FNR: False-negative rate
OSNA: One-step nuclei acid amplification
ypT0: Pathologic complete response in breast
ypTis: Pathologic residual in situ carcinoma in breast
ER: Estrogen receptor
PR: Progesterone receptor
IPTW: Inverse probability of treatment weighting
CI: Confidence intervals
HR: Hazard ratios
SI: Supplementary Information
NS: No statistical significance
