# Supplementary material for: Sentinel lymph node biopsy before and after neoadjuvant chemotherapy in cN0 breast cancer patients: impact on axillary morbidity and survival—a propensity score cohort study
Source: Breast Cancer Res Treat. 2024 Apr 18;206(1):131–41. doi: 10.1007/s10549-024-07274-1 (PMC11182812; doi:10.1007/s10549-024-07274-1)

SUPPLEMENTARY INFORMATION (SI)

**Figure SI-1**. Pathological response to NACT.


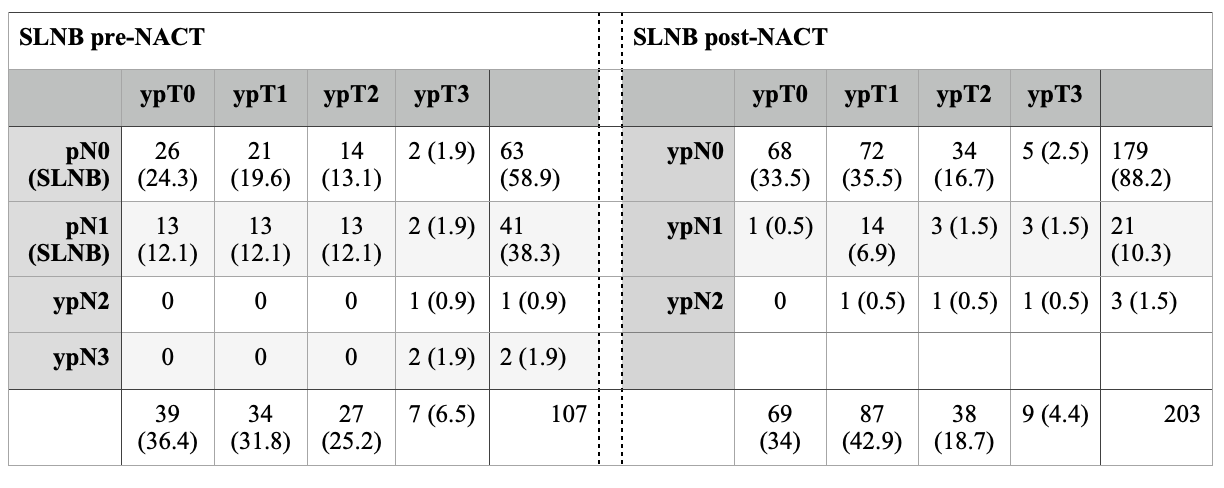


**Table SI-1.** Sentinel Lymph node results according breast cancer subtype in pre-NACT cohort.

|  | **hormone receptor +**  **her2 –**  **(n=28)** | **hormone receptor +**  **her2 + (n=23)** | **her-2 pure (n=21)** | **triple negative (n=35)** | **Total (n=107)** |
| --- | --- | --- | --- | --- | --- |
| **Pre-NACT SLN (107)** | N=28 | N=23 | N=21 | N=35 | N=107 |
| **SLN identification rate (%)** | 28 (100) | 23 (100) | 21 (100) | 35 (100) | 107 (100) |
| **Patients with SLN positive (%)** | 16 (57.1) | 13 (56.5) | 8 (38.1) | 8 (22.8) | 45 (42.1) |
| **macrometastases (%)** | 10 (62.5) | 13 (100) | 3 (37.5) | 5 (62.5) | 31 (68.9) |
| **micrometastases (%)** | 6 ( 37.5) | 0 | 5 (62.5) | 3 (37.5) | 14 (32.1) |
| **Lymphadenectomy (%)** | 11 (39.3) | 13 (56.5) | 3 (14.3) | 5 (14.3) | 32 (29.9) |
| **no residual disease (%)** | 5 (45.5) | 11 (84.6) | 3 (100) | 2 (40) | 21 (65.6) |
|  |  |  |  |  |  |
| **Post-NACT SLN (203)** | N=64 | N=49 | N=17 | N=73 | N=203 |
| **SLN identification rate (%)** | 63 (98.4) | 48 (97.9) | 16 (94.1) | 71 (97.2) | 107 (100) |
| **Patients with SLN positive (%)** | 13 (20.6) | 7 (14.5) | 0 | 5 (7) | 25 (12.5) |
| **macrometastases (%)** | 7 (53.8) | 2 (28.6) | 0 | 2 (28.6) | 11 (44) |
| **micrometastases (%)** | 6 ( 46.1) | 5 (71.4) | 0 | 3 (60) | 14 (46) |
| **Lymphadenectomy (%)** | 8 (12.5) | 2 (4.1) | 1 (0.5) | 4 (2) | 15 (7.4) |
| **no residual disease (%)** | 3 (37.5) | 0 | 1 (100) | 4 (100) | 8 (5.3) |
|  |  |  |  |  |  |

**Figure SI-2.** Disease-free survival according results of SLNB in pre-NACT and post-NACT group.


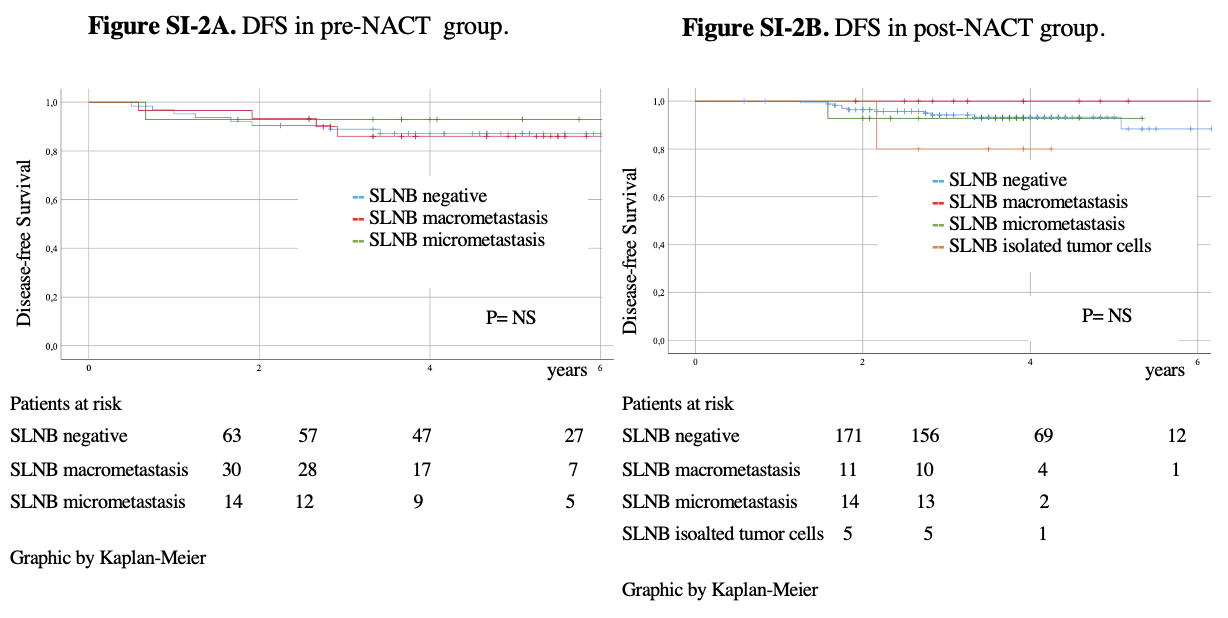


Figure SI-3. Overall Survival (Survival) and Disease-free survival in original data and after IPTW after the exclusion of patients who received Capecitabine and/or Pertuzumab.


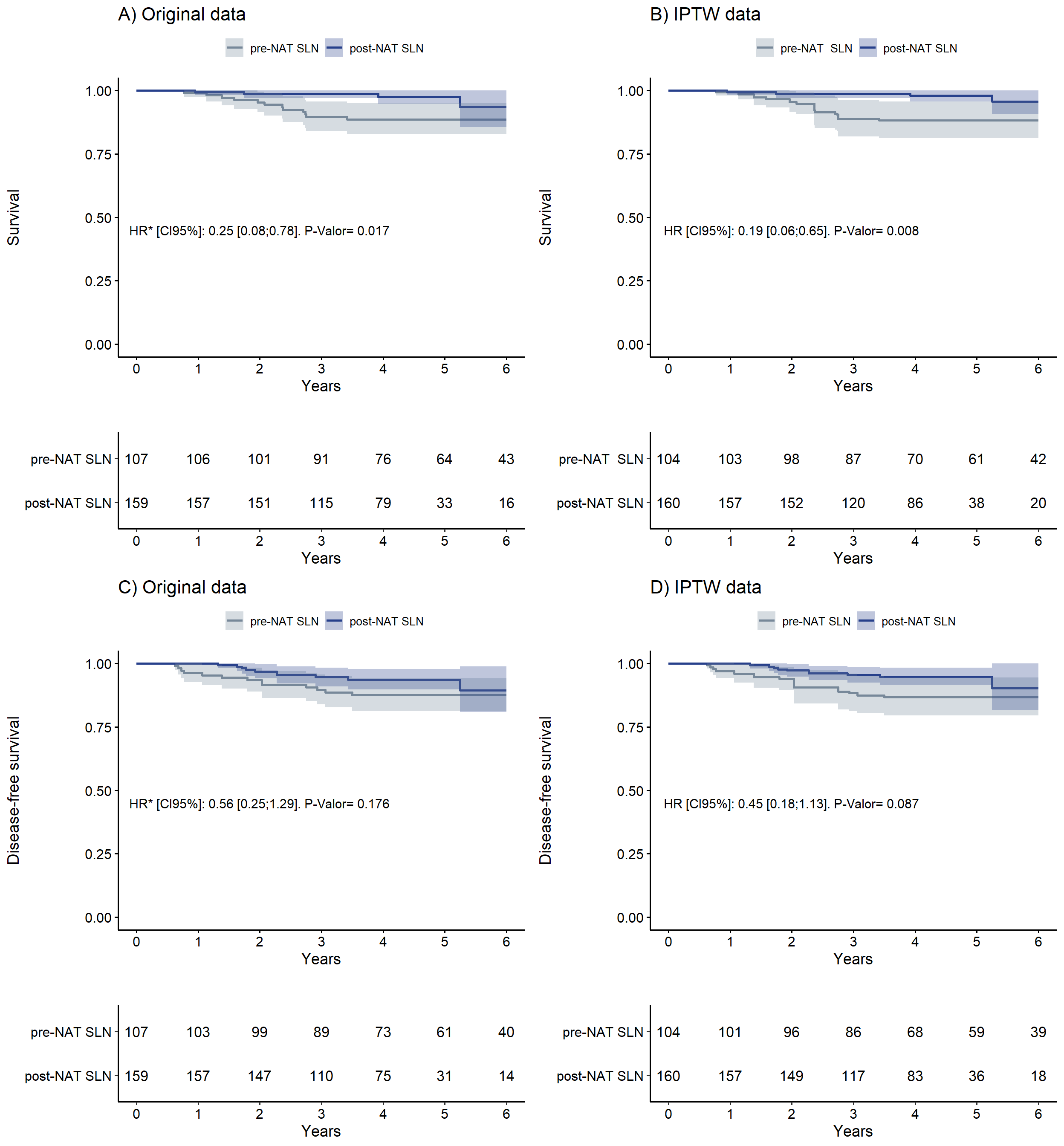

Supplement: Supplementary file 1 — Supplementary file1 (DOCX 309 kb) [file 10549_2024_7274_MOESM1_ESM.docx]
